# A Machine Learning-Based Modeling Approach for Dye Removal Using Modified Natural Adsorbents

**DOI:** 10.1021/acs.jcim.5c01016

**Published:** 2025-08-08

**Authors:** Betul Uzbas, Suheyla Kocaman

**Affiliations:** † Computer Engineering Department, 531804 Konya Technical University, 22250 Konya, Turkey; ‡ Chemical Engineering Department, 531804 Konya Technical University, 22250 Konya, Turkey

## Abstract

This study used machine learning models to investigate the potential of biosorbents derived from natural fruit seed waste (apricot, almond, and walnut) for removing a cationic dye. Levulinic acid (LA)-modified powders of almond shell (ASh), apricot kernel shell (APKSh), and walnut shell (WSh) were used to remove methylene blue (MB) from an aqueous solution, producing 105 experimental data points under various circumstances. Attributes included pH (3–5), adsorbent dose (0.4–6.0 g/L), concentration (10–500 mg/L), time (30–600 min), and temperature (25–55 °C). Species information was incorporated into the data set using the One-Hot Encoding method. The data were normalized using the min-max method, and due to the non-normal distribution of the data, Spearman correlation analysis was employed to rank the importance of the attributes. Gradient Boosting (GB), Multilayer Perceptron (MLP), XGBoost (XGB), and Random Forest (RF) algorithms were applied for regression estimation. Based on 5-fold cross-validation results, the GB model achieved the highest performance, with R^2^ values of 0.8858 for removal percentage and 0.9532 for adsorption capacity.

## Graphical abstract

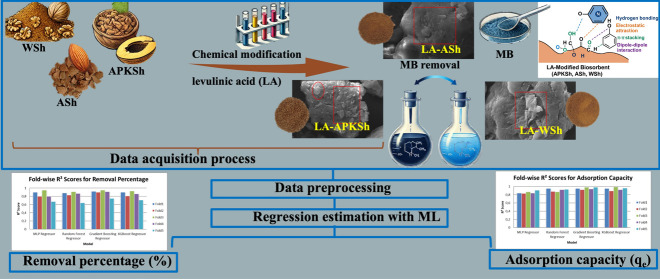

## Introduction

Discharging chemical pollutants into water bodies due to industrial activities, particularly within the chemical and textile sectors, is a critical environmental issue today. These pollutants encompass many substances, including organic compounds, heavy metals, and synthetic dyes. Among them, dyes used extensively in the textile industry are of particular concern due to their structural complexity and persistence. Structurally, dyes are aromatic compounds containing functional groups such as azo, anthraquinone, methyl, carbonyl, nitro, and aryl methane.^1^ Most modern dyes are synthetic and derived from petroleum, making them highly resistant to natural bio­degradation. As early as the 19th century, awareness grew regarding the hazardous effects of synthetic dyes, including their toxicity and carcinogenic potential, which spurred efforts to develop effective wastewater treatment strategies.^2^ Nevertheless, despite these known environmental risks, the use of synthetic dyes remains widespread due to their vibrant color range, year-round availability, and ease of industrial application.

The removal of dyes from wastewater has been investigated using a range of treatment methods, such as coagulation–flocculation,^3^ membrane filtration,^4^ advanced oxidation processes,^5^ biological treatments,^6–8^ chemical oxidation,^9^ and adsorption.^10–12^ Because of its ease of use, adaptability, nontoxicity, and controllable parameters, adsorption has become one of the most popular and effective techniques among them.^5^

Natural materials—such as lignocellulosic and plant-based wastes, clays, biopolymers, biocomposites, and activated carbons derived from biomass—are commonly used as adsorbents due to their eco-friendly origin.^13^ Numerous investigations have assessed the performance of these materials in removing various hazardous dyes from wastewater.^14,15^ For instance, agricultural residues like fruit stones and peels have been pyrolyzed to produce activated carbon for decolorization.^16^ However, the production process can be cost-prohibitive and often leads to challenges in replacing and disposing of the spent carbon.^17^ Consequently, research has increasingly focused on directly applying inexpensive, readily available waste materials for dye adsorption. Agricultural byproducts, in particular, offer a promising and sustainable alternative to synthetic adsorbents owing to their availability, low cost, and environmental safety.^18^ Notable examples of such bioadsorbents include cashew,^19^ *Terminalia chebula*,^20^ almond, walnut, and apricot shells,^10,11^ cherry kernel,^21^ peanut^22^ peach kernel shell,^23^ pistachio,^24^ and coconut,^25^ all of which have been successfully utilized in treating dye-laden wastewater.

In recent years, the application of machine learning (ML) techniques has expanded significantly across various scientific domains, including chemistry and environmental engineering. For instance, Shomope et al.^26^ created a multilayer perceptron-based artificial neural network (ANN) to forecast biohydrogen production from organic waste materials. The model was trained using 180 data points collected from 35 different investigations, with the output being hydrogen yield and the inputs being substrate and inoculum type, concentration, pH, and temperature. The model demonstrated strong performance, achieving an R^2^ value of 0.8381 through 5-fold cross-validation. Similarly, Mahata et al.^27^ investigated biohydrogen production via dark fermentation and compared the effectiveness of various ML approaches, including ANN, Support Vector Machines (SVM), and response surface methodology. Among these, SVM delivered the best predictive accuracy with an R^2^ of 0,988. In another study, Hosseinzadeh et al.^28^ employed Gradient Boosting (GB), SVM, Random Forest (RF), and AdaBoost algorithms for modeling hydrogen production, achieving R^2^ scores of 0.893, 0.885, 0.902, and 0.889, respectively.

Further contributions by Shomope et al.^29^ focused on hydrogen generation in proton exchange membrane (PEM) electrolysis systems. Random Forest and XGBoost algorithms yielded exceptional prediction accuracy, with R^2^ values of 0.9898 and 0.9894. Similarly, Bilgiç et al.^30^ applied an ANN model to examine the effects of parameters such as magnetic field strength, electrode material, electrolyte type, temperature, and time on hydrogen production in water electrolysis systems, reporting a high correlation (R = 0.973) between model outputs and experimental data. Odabaşı et al.^31^ utilized ML techniques to assess and predict key performance indicators of reverse osmosis (RO) membranes in municipal wastewater recovery, identifying ANN as the most accurate for pressure prediction, RF for salt passage, and multiple linear regression for permeate flow rate.

AI models can accurately predict the effectiveness of adsorbents in removing pollutants from wastewater. They have great potential in water treatment thanks to hybrid systems with appropriate data integration.^32^ ML models have been employed in literature to predict the performance of adsorbent materials in dye removal. In the study conducted by Liu et al.^33^ to predict the adsorption capacity of hydrochar on different dyes, the GB model was the most successful model, with an R^2^ value of 0.9629 being reported. The most effective variable in the feature importance analysis was the experimental conditions. In the study conducted by Hamri et al.^34^ with Kaolinite (DD3) and its acid-treated form, the GPR-PSO (Gaussian Process Regression–Particle Swarm Optimization) model yielded the most optimal result; R^2^ = 0.9978 was obtained. The highest adsorption was observed at a pH of 11. In the study of Gamboa et al.,^35^ the GB model was combined with Bayesian optimization in the removal of Congo red using biochar (ABHC), with the best result being obtained with approximately 90.47% efficiency. Rajput et al.^36^ found that, in their study on methylene blue removal, the RF model was the most successful. They achieved the highest accuracy with R^2^ = 0.94. The most effective variable was determined to be the initial dye concentration (*C*_0_). In the study by Kulkarni et al.,^37^ the XGBoost model demonstrated the optimal performance in the context of engineered carbon systems (ECS), with an R^2^ value of 0.978 being reported. The most effective factors are identified as dye concentration, ECS dose and pH. The findings of these studies demonstrate the potential of machine learning to generate precise predictions in water treatment processes involving various adsorbents and dyes, thereby contributing to environmental sustainability. In studies by Kumari et al.,^38^ methylene blue (MB) and crystal violet (CV) dyes were removed with over 98% efficiency using Saccharum officinarum L., and the artificial neural network (ANN) model showed the highest prediction accuracy with R^2^ = 0.9236. In the research conducted by Kumari et al.,^39^ a modeling approach and machine learning (ML) techniques were employed to remediate an organic pollutant using a *Juglans regia* adsorbent. The process was successfully predicted with artificial neural networks (ANNs) (R^2^ = 0.9373) and the RSM (R^2^ = 0.9117) models, and the adsorption was spontaneous and exothermic.

The present study integrates both batch and continuous systems to investigate novel biosorbent-based strategies for dye removal from wastewater. The apricot, almond, and walnut shells employed herein are commonly available agricultural residues in Turkey.^40^ These natural materials are rich in hydroxyl functional groups, which facilitate the adsorption of pollutants and can be further functionalized by introducing sulpho, amino, or carboxyl groups.^41^ However, their inherent adsorption capacity is often limited. Studies have shown that chemical modification can significantly improve the efficiency of these biosorbents by enhancing their surface reactivity and functional group density.^41–43^

The adsorbent’s performance is a crucial consideration in the adsorption process design field, typically evaluated through adsorption capacity assessments.^44^ This study uses data mining and machine learning methodologies to predict the target variables, removal percentage (% R), and adsorption capacity (mg/g), and rank the importance of various attributes affecting these variables. The key contributions of this study to the existing literature are as follows:

- The use of agricultural byproducts such as apricot kernel shells (APKSh), almond shells (ASh), and walnut shells (WSh), abundant in Turkey, presents a sustainable and eco-friendly approach to waste management, promoting the effective use of domestic, low-cost biosorbent resources.
- This study proposes a novel method for improving the performance of biosorbents by chemically treating natural shell wastes, thereby enhancing their adsorption capacity—a subject not previously explored in the literature.
- This research integrates batch and continuous systems, allowing for development of more comprehensive and practical solutions for dye removal.
- The application of data mining and machine learning techniques in the adsorption process enables the automated prediction of target variables (removal percentage and adsorption capacity) and identification of the most effective parameters. This advancement contributes to developing digital decision support systems for process optimization.

## Methodology

### Adsorption Studies and Data Acquisition Process

The data sets for this study were obtained from the peer-reviewed research article of Kocaman in 2020.^10^ A simple dye called MB was used as an adsorbate. A nitrogen center in its aromatic ring gives MB, a cationic dye, a positively charged surface. Adsorption tests were conducted on modified natural adsorbents derived from biomass waste (apricot kernel shells (APKSh), almond shells (ASh), and walnut shells (WSh)) modified with levulinic acid (LA). A series of aqueous dye solutions was prepared, varying in concentration from 10 to 500 mg/L, employing distilled water as the solvent. A volume of 25 mL of each solution was treated in a shaking bath containing known amounts of adsorbent (0.4–6 g/L) at a speed of 150 rpm Following a designated period (0–10 h), the mixture was centrifuged at 5000 rpm for 5 min to separate the liquid and solid phases. A UV–vis spectrophotometer was used to analyze the aqueous phase for methylene blue at a maximum wavelength of 661 nm, corresponding to the compound’s highest absorption peak (λ_max_). The amount of adsorbed dye was ascertained by computing the difference between the dye solution’s initial and final concentrations. Eqs 1 and 2 were used to calculate the adsorption % and the adsorbent’s capacity, as explained below.

1 $$\%R=\frac{C_{0}-C_{e}}{C_{0}}\times 100$$

2 $$q_{e}=\frac{C_{0}-C_{e}}{m}\times V$$

*C*_0_: initial dye concentration (mg/L)
*C*_*e*_: final dye concentration (mg/L)
*R*: removal percentage (%)
*q*_*e*_: adsorbent capacity (mg/g)
*V*: volume of dye solution (L)
*M*: amount of adsorbent (g)

The present study investigates the effects of pH (3, 5, 7, 8, 10), adsorbent dose (0.4, 1, 2, 4, 6 g/L), temperature (25, 35, 45, 55 °C), time (30, 60, 120, 180, 240, 240, 300, 360, 420, 480, 540, 600 min) and concentration (10, 20, 30, 40, 50, 100, 150, 200, 300, 500 mg/L) on MB adsorption. The initial pH effect is examined first in this study. Then, the effects of adsorbent dose, concentration, time, and temperature on removal percentage (%) and adsorption capacity (*q*_*e*_) are examined. Consequently, a data set comprising 105 samples was obtained. The parameters of the LA-modified biosorbent (0.025 L; 250 rpm) utilized in the investigation of the effects of various factors on MB adsorption are presented in Table 1.

**Table 1:** LA-Modified Biosorbents Parameters Used to Explore the Effects of Various Factors on MB Adsorption (0.025 L; 250 rpm)

	pH	Dosage (g/L)	Concn (mg/L)	Time (min)	Temp (°C)
Effect of pH	3, 5, 7, 8, 10	1.0	10	300	25
Effect of dosage	5	0.4, 1.0, 2.0, 4.0, 6.0	10	300	25
Effect of MB concentration	5	2.0	10, 20, 30, 40, 50, 100, 150, 200, 300, 500	300	25
Effect of time	5	2.0	100	30, 60, 120, 180, 240, 300, 360, 420, 480, 540, 600	25
Effect of temperature	5	2.0	100	300	25, 35, 45, 55

### Data Preprocessing

The data set used in this investigation includes maximum removal percentage (%) and adsorption capacity (*q*_*e*_) values obtained by removing MB dye from water using different adsorbent types. These adsorbents were modified by incorporating LA into almond, walnut, and apricot kernel powders. The data set under consideration encompasses pH, adsorbent dose, concentration, temperature, and time values.

One-hot encoding is a prevalent technique for the digitization of categorical data. This method creates a separate binary column (0 or 1) for each category in the attribute to be digitized. Creating new attribute values of the type categorical value, consisting of three attribute values, was performed for almond, walnut, and apricot kernels. This was achieved employing One-Hot Encoding. Consequently, a data set consisting of 105 samples with eight attributes and two target values was created. The values and descriptions of the data set attributes are presented in tabular form in Table 2. The categorical feature type comprised three distinct material types (almond, walnut, and apricot kernel) that could affect the adsorption behavior in terms of physicochemical properties and surface properties. One-hot encoding was adopted to ensure that the model could capture and distinguish potential variation in adsorption performance attributable to these types. This transformation resulted in a marginal increase in the dimensionality of the data set (from one to three binary variables) without any adverse impact on model performance, as evidenced by the findings of the cross-validation process. Given the limited number of categories and observations, the impact on model complexity was minimal, and the benefit of capturing material-specific effects justified the use of this encoding strategy.

**Table 2:** Dataset Attribute Values and Descriptions

Attribute	Description	Value
Almond	If almond is present, the result will be 1; if not, it will be 0.	0 or 1
Walnut	If walnut is present, the result will be 1; if not, it will be 0.	0 or 1
Apricot kernel	If apricot kernel is present, the result will be 1; if not, it will be 0.	0 or 1
pH	MB solution pH	3, 5, 7, 9, 10
Adsorbent Dose (g/L)	Amount of natural adsorbent	0.4, 1.0, 2.0, 4.0, 6.0
Concn (mg/L)	Concentration of MB	10, 20, 30, 40, 50, 100, 150, 200, 300, 500
Temp (°C)	Ambient temperature	25, 35, 45, 55
Time (s)	Adsorption time	30, 60, 120, 180, 240, 300, 360, 420, 480, 540, 600
Removal percentage (%)	Target 1	[22.7, 98.83]
Adsorption capacity (mg/g)	Target 2	[1.63, 99.4]

During data analysis, it is important to note that the value range of each attribute may vary, with the potential to impact the analysis results. To eliminate such interactions, it is necessary to normalize or standardize the data. This process enables the data to be transformed into a specific range, such as [−1,1] or [0,1].^45^ In this study, Min-Max normalization is employed for each attribute, with the data being rescaled such that the minimum value is set to 0 and the maximum value is set to 1.

### Statistical Analysis

In data analysis, it is imperative to thoroughly examine the data distribution to select the most appropriate statistical methods. The Kolmogorov–Smirnov (KS) test measures the differences between two data groups by comparing their distribution functions.^46,47^ According to the data distribution characteristics, the analysis process is shaped by applying parametric or nonparametric tests.

Correlation analysis is a method of determining the direction and strength of the relationship between variables. The Pearson Correlation Coefficient^48^ should be preferred when the data are normally distributed. Conversely, if the data do not demonstrate a normal distribution, the Spearman^49^ correlation coefficient should be considered a more appropriate method.

### Machine Learning

The capacity of computers to acquire knowledge has the potential to generate software applications that can enhance their functionality with experience. In this field, ML algorithms have achieved significant success in applications such as data mining in many different fields. Its extensive application encompasses domains like extracting valuable information from substantial data sets and decision support systems.^50^ Within machine learning, classification and regression represent fundamental methodologies that seek to predict future outcomes through analyzing existing data. The primary function of classification is to predict categorical labels, whereas regression is employed to predict continuous numerical values. Regression is a predictive modeling method used when the target variable is continuous. Regression analysis is widely utilized for determining numerical predictions and distributional trends. Before classification and regression, relevance analysis can be performed to identify important attributes, and unnecessary attributes can be removed from the process.^45^

It has been demonstrated that neural network learning methods effectively learn functions with continuous and discrete values. Furthermore, these methods are robust to noise in the training data. The backpropagation algorithm is one of this field’s most widely utilized methods. This algorithm generates hypotheses by updating the weights within a given network architecture, enabling the model to learn by minimizing the error rate with the gradient descent method. Multilayer feed-forward (MLP) artificial neural networks have been shown to possess the capacity to approximate any function with a certain level of accuracy, provided that they contain a sufficient number of neurons. A significant benefit of the back-propagation algorithm is its capacity to identify novel features that are not inherently present in the input data but emerge during the learning process.^50^

Ensemble methods aim to obtain more robust predictions by combining simple models. It is acknowledged that weak learners may not achieve optimal results independently; however, their efficacy can be enhanced through collaborative efforts. Bagging,^51^ RF,^52^ and Boosting are popular ensemble methods. The process of bagging trains involves the construction of multiple decision trees, each derived from a different bootstrapped data set sample. This approach reduces variance by averaging the predictions made by these trees. RF represents an enhancement of Bagging, whereby the interdependence between trees is diminished and the model’s generalization capability is augmented by employing a specific number of randomly selected variables at each split.^53^

The objective of boosting algorithms is to construct a more robust model through the successive training of weak learners. Each new model incorporates improvements derived from the errors observed in previous iterations, thereby enhancing the model’s accuracy through refinement and reduction in error rate. The gradient boosting method, developed for regression problems, focuses similarly, minimizing the loss function at each step and gradually reducing the model’s prediction errors.^54^ A gradient boosting decision tree ensemble designed for great scalability and efficiency is called XGBoost.^55^ By reducing the loss function, XGBoost produces an additive expansion of the objective function, which is comparable to the gradient boosting method. This procedure makes it easier to produce more reliable predictions.

### Evaluation Metrics

#### k-Fold Cross Validation

Resampling methods are widely used in statistical analysis and can often be computationally expensive. Using various subsets of the training data, these approaches repeatedly apply the same statistical procedure. However, thanks to improved computational capacities, the computational requirements of these methods are no longer a significant obstacle. Cross-validation (CV) assesses model accuracy or determines appropriate model flexibility. In particular, k-fold CV divides the data set into k equal groups, using a different group as a validation set each time, and calculates the model’s accuracy. This process is repeated k times to obtain error estimates based on each validation set, and the final model error is calculated by averaging them.^53^

#### RMSE and MAE

The predictive performance of ML models is determined by two error metrics: the mean absolute error (MAE) and the root-mean-square error (RMSE), which are widely used in regression analysis. These metrics quantitatively reveal the proximity of the model’s predictions to the true values and are widely preferred in the literature with regard to interpretability.

MAE represents the average of the absolute differences between the predicted and true values, while RMSE is calculated by taking the square root of the mean square of the prediction errors^56^ (eq 3).

3 $$\begin{array}{l} \mathrm{MAE}=\frac{1}{n}\overset{n}{\underset{i=1}{\sum }}|yi-f\left(xi\right)|\\ \mathrm{RMSE}=\sqrt{\frac{1}{n}\overset{n}{\underset{i=1}{\sum }}{\left(yi-f\left(xi\right)\right)}^{2}} \end{array}$$

where *yi* is the true (observed) dependent variable value and *f*(*xi*) is the dependent variable value predicted by the regression model.

#### R^2^ Score

The primary purpose of regression is to create a model that provides the best fit to the input data and minimizes the amount of error. To evaluate the performance of the regression model, measures such as total sum of squares (SST) and regression error squared (RMS) are used:^57^

- **SST (Total Sum of Squares):** expresses the amount of error in the predictions made using the overall mean of the dependent variable (eq 4).
4 $$\mathrm{SST}=\underset{i}{\sum }{\left(yi-\mathrm{y̅}\right)}^{2}$$
- **SSM (Regression Sum of Squares):** expresses the amount of error created when the model’s dependent variable is on (eq 5).
5 $$\mathrm{SSM}=\underset{i}{\sum }{\left(f\left(xi\right)-\mathrm{y̅}\right)}^{2}$$

In eqs 4 and 5, *yi* is the true (observed) dependent variable value, *y̅* is the mean value of the dependent variable, and *f*(*xi*) is the dependent variable value predicted by the regression model.

R^2^ (Coefficient of Determination) is used to determine the success of the regression model. The R^2^ value varies between 0 and 1 and shows how well the model explains the dependent variable (eq 6):

6 $$R^{2}=\frac{\mathrm{SSM}}{\mathrm{SST}}$$

## Proposed Framework

The proposed framework is presented in Figure 1. As illustrated in Figure 1, the summary of the study is as follows: First, in the Data Acquisition Process step, apricot, almond, and walnut shell powders, which are natural fruit seed wastes, were first ground and sieved on 53 μm sieves to become powder. Natural shells that had undergone a chemical modification with LA were utilized as adsorbents in an innovative and environmentally friendly approach to remove MB dye.^10^ The present study examined the adsorption behavior of MB on biosorbents, with a range of parameters investigated, including the pH (3–10), sorbent dose (0.4–6 g/L), initial dye concentration (10–500 mg/L), and temperature (25–65 °C). The adsorbents’ maximum adsorption capacity (mg/g) and removal percentage (%) values were determined for each case. The data set under consideration consists of 35 separate samples for all almond, walnut, and apricot seed adsorbents, a total of 105 samples. The attribute values consist of the following: pH, sorbent dose, concentration, time, and temperature. According to the Kolmogorov–Smirnov test, it was determined that the data did not demonstrate a normal distribution. The subsequent determination of the importance order of the attributes was achieved by employing Spearman’s correlation coefficient between the target variables of removal percentage (%) and adsorption capacity (mg/g).

**Figure 1:**
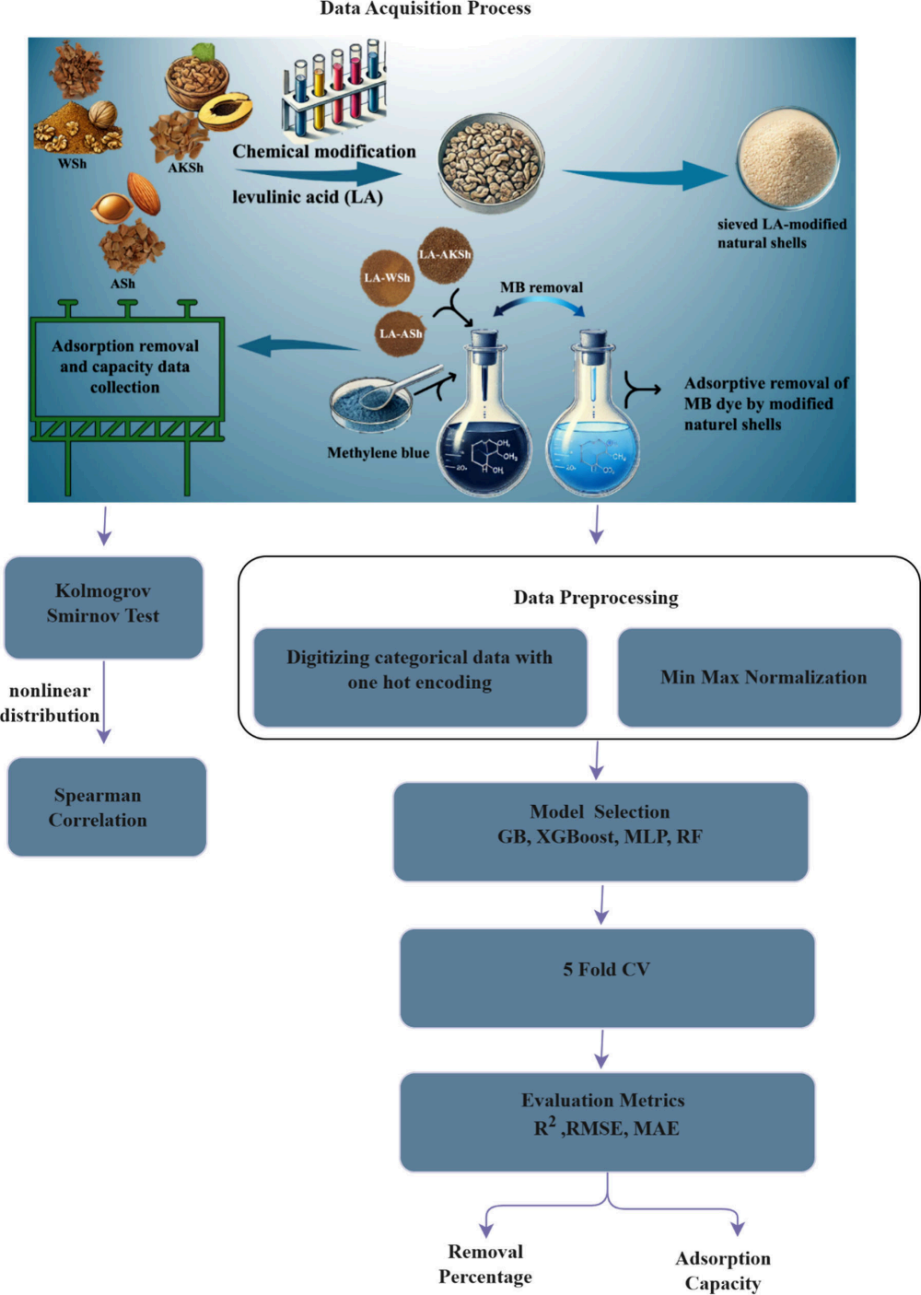
Data acquisition and ML processing workflow.

Figure 2 demonstrates that the hierarchy of significance is established by the correlation coefficient among the target adsorption capacity values, the removal percentage, and the characteristics. The Species attribute was also added to the Data Set using One Hot Encoding. A new column value was created for each species, such that if the sample belonged to that species, it would be assigned a value of 1. Otherwise, it would be assigned a value of 0. Consequently, three additional columns were added for species values, resulting in a data set with a total of eight attributes and two target values. The MinMax normalization process was employed for standardization, with all attribute values ranging from 0 to 1. The present study employed various popular ML models for regression estimation, including GB, XGB, MLP, and RF methods. The MLmodels utilized in the study were configured with hyperparameters that are predominantly favored in the extant literature and closely resemble the library defaults, with a view to mitigating the risk of overfitting in view of the limited data set. The evaluation of the model was conducted through the utilization of 5-fold cross-validation. In this study, the MLP model was constructed with two hidden layers comprising 10 and 20 neurons, respectively. The rectified linear unit (ReLU) activation function and the ‘lbfgs’ solver were utilized. The RF model was configured with 100 trees, with the number of estimators set to 100. The GB and XGBoost models were both constructed with 100 trees and a learning rate of 0.1. Tables 3 and 4 illustrate the R^2^ performance scores achieved by the models across each fold of the 5-fold cross-validation process.

**Figure 2:**
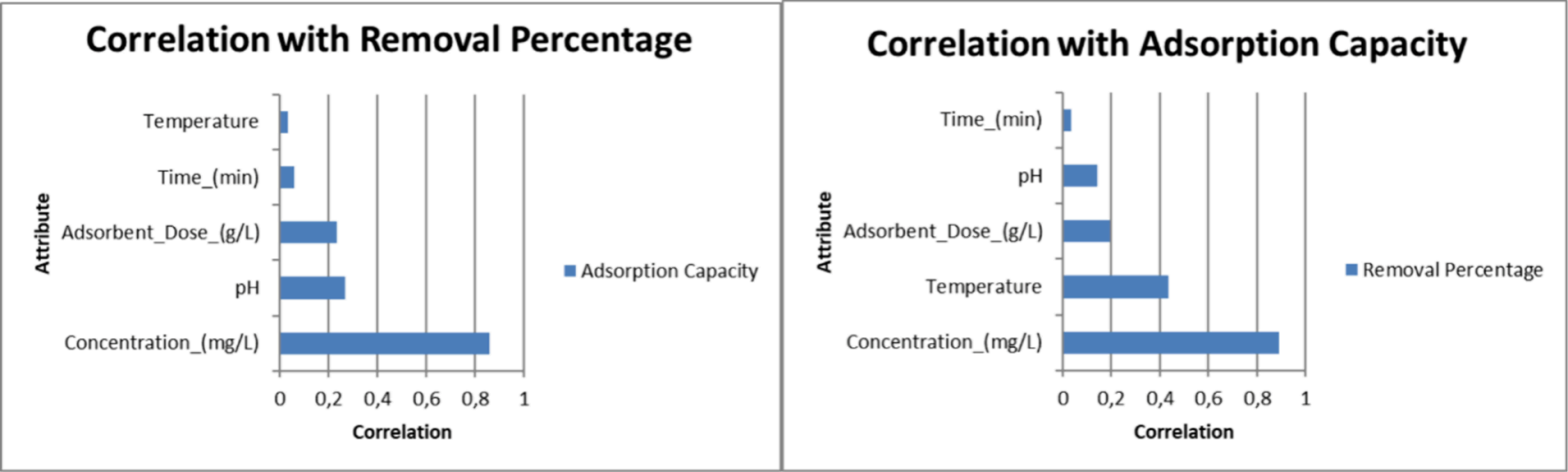
Correlation values between target values and attributes.

**Table 3:** Results of the Regression Estimation Process for the Removal Percentage

Model	R^2^	RMSE	MAE
MLP	0.8041	5.0713	3.5355
RF	0.8268	5.0891	3.3009
GB	0.8858	4.0837	2.6381
XGBoost	0.8423	4.7345	3.0092

**Table 4:** Results of the Regression Estimation Process for Adsorption Capacity

Model	R^2^	RMSE	MAE
MLP	0.8844	8.4585	4.9902
RF	0.9086	7.0426	3.9303
GB	0.9532	5.3473	2.8165
XGBoost	0.9424	5.9737	3.0242

The performance of the regression models was evaluated using the R^2^, RMSE and MAE metrics. The model results for removal percentage are presented in Table 3 and the results for adsorption capacity in Table 4. The analyses show that the Gradient Boosting (GB) model achieved the highest R^2^ values for both target variables in particular (0.8858 for removal percentage and 0.9532 for adsorption capacity). These high R^2^ scores indicate that the models successfully explain the variance of the relevant target variables and demonstrate strong predictive ability. However, the absolute magnitudes of the RMSE and MAE values seem higher than in some previous studies. The main reason for this is that these metrics are unit-dependent; therefore, the value ranges of the target variables should be considered when making direct comparisons. Studies in the literature where error metrics are reported as low are generally based on data sets where the target variable is limited to a narrow range (e.g., 90–100). Error metrics naturally remain low within such limited ranges. In this study, removal percentage varies over a wide range, from 22.7 to 98.83 mg/g, and adsorption capacity from 1.63 to 99.4%. These wide scales inevitably increase the magnitude of the RMSE and MAE values. Therefore, when interpreting error metrics, it is important to consider the absolute values and distribution ranges of the target variables. In this context, high R^2^ scores strongly indicate that the models have high predictive power, regardless of the absolute error magnitudes.

Figures 3 and 4 illustrate the R^2^ performance scores achieved by the models across each fold of the 5-fold cross-validation process.

**Figure 3:**
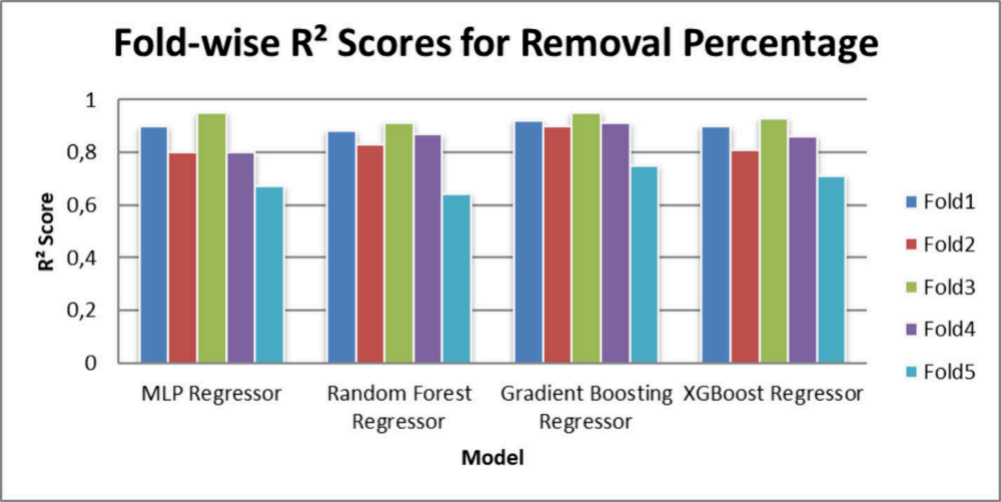
Fold-wise R^2^ scores for removal percentage.

**Figure 4:**
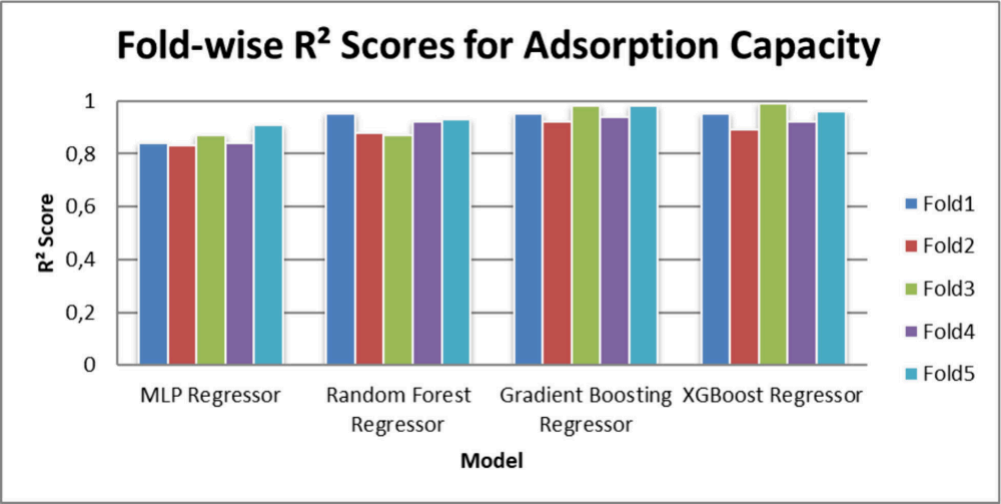
Fold-wise R^2^ scores for adsorption capacity.

In Table 5, the average R^2^ score obtained via 5-fold cross-validation is presented.

**Table 5:** Results of the Regression Estimation Process

Model	R^2^ Score for Removal Percentage	R^2^ Score for Adsorption Capacity
GB	0.8858	0.9532
XGBoost	0.8423	0.9424
RF	0.8268	0.9086
MLP	0.8003	0.8614

## Results and Conclusions

### Surface Morphology and Adsorption Mechanism

Surface morphology analyses of raw and LA-modified biomass powders were carried out using scanning electron microscopy (SEM) (Figure 5). The untreated ASh (Figure 5a1) exhibited a relatively compact and fibrous structure with limited surface irregularities and pore development, indicating a low number of accessible adsorption sites for dye molecules. In contrast, the LA-treated ASh (Figure 5a2) demonstrated clear surface disruption, with visible microcracks, flaking, and increased textural heterogeneity, suggesting an enhanced availability of active sites for adsorption due to surface oxidation and partial depolymerization of lignocellulose components.^11,58^

**Figure 5:**
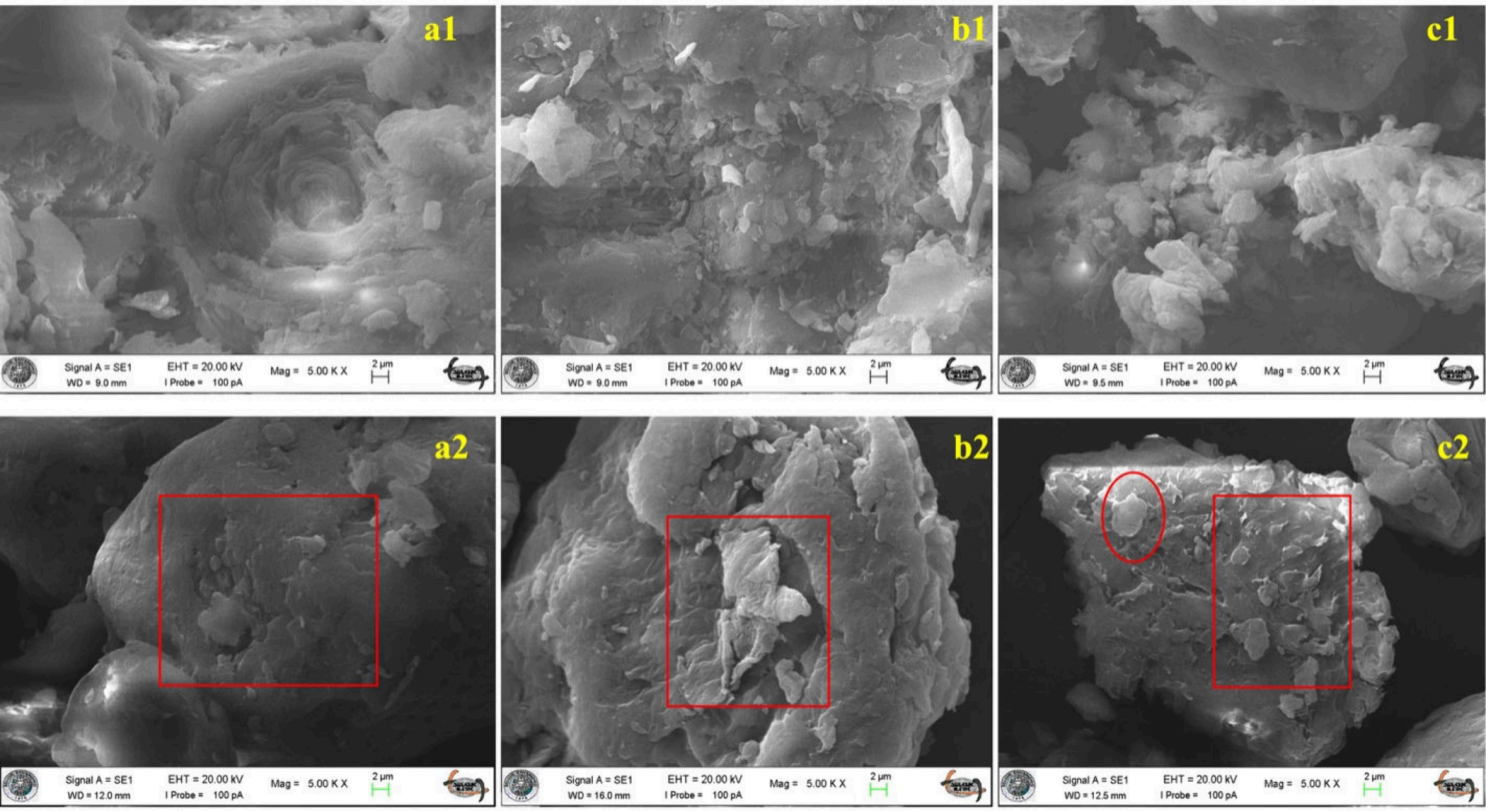
SEM images of raw and LA-modified biomass powders: (a1) raw ASh, (b1) raw WSh, (c1) raw APKSh; (a2) LA-modified ASh, (b2) LA-modified WSh, (c2) LA-modified APKSh (Mag = 5.00 KX, 2 μm).

Similarly, the raw WSh (Figure 5b1) showed a dense and fragmented surface structure with limited porosity and a comparatively smoother texture. However, following LA treatment (Figure 5b2), the surface displayed distinct stratification, roughness, and delamination, implying a more developed porous network and increased surface reactivity. These morphological changes are likely attributed to esterification or hydrogen bonding between LA and the hydroxyl groups on the lignocellulosic matrix, which disturb the original structure and create accessible binding pockets.^59^

The APKSh (Figure 5c1) before modification presented a compact, plate-like morphology with minimal surface irregularities. Post-treatment with LA (Figure 5c2), the structure was noticeably more disrupted, with visible fragmentation, porous zones, and increased surface heterogeneity. These features point toward enhanced dye accessibility and the formation of polar functional groups such as carboxyl and carbonyl moieties on the surface, which are favorable for electrostatic and π–π interactions with aromatic dye molecules like MB.^60^

Overall, the SEM images confirm that LA modification induces significant changes in surface morphology, increasing porosity and roughness across all biomass types examined. This enhancement is consistent with previous findings in the literature, where LA-treated lignocellulosic materials exhibited improved adsorption capacities for organic pollutants due to chemical activation and surface restructuring.^58,10^

Levulinic acid (CH_3_C­(O)­CH_2_CH_2_COOH) is a bifunctional organic acid containing both a ketone and a carboxylic acid group.^60^ During the modification process, the carboxylic acid group of LA is capable of forming ester or hydrogen bonds with the hydroxyl (−OH) groups abundantly present on the lignocellulosic structure of the fruit shell powders (APKSh, ASh, WSh).^58,61^ The interaction is primarily via esterification or surface grafting, especially under mild heating or catalytic conditions.^62^

This surface modification introduces additional polar oxygen-containing groups (e.g., carbonyl, carboxyl, hydroxyl) onto the adsorbent surface, thereby^58,61,62^

- increasing the number of active binding sites for dye molecules,
- enhancing surface acidity, which improves electrostatic attraction toward cationic dyes like MB,
- improving hydrogen bonding interactions between MB and the modified surface, and
- slightly increasing the hydrophilicity and swelling behavior, thus improving dye diffusion into surface pores.

Furthermore, the presence of LA-derived carbonyl groups facilitates π–π stacking and dipole–dipole interactions with the aromatic structure of MB, thereby enhancing the dye’s retention.

This mechanistic understanding is consistent with prior findings in the literature, where LA-modified lignocellulosic adsorbents showed enhanced removal capacities for various organic pollutants due to increased surface polarity, electronic interactions, and binding affinity.^58,61,62^ The proposed mechanism for the interaction of MB dye onto LA-based adsorbents is provided in Figure 6.

**Figure 6:**
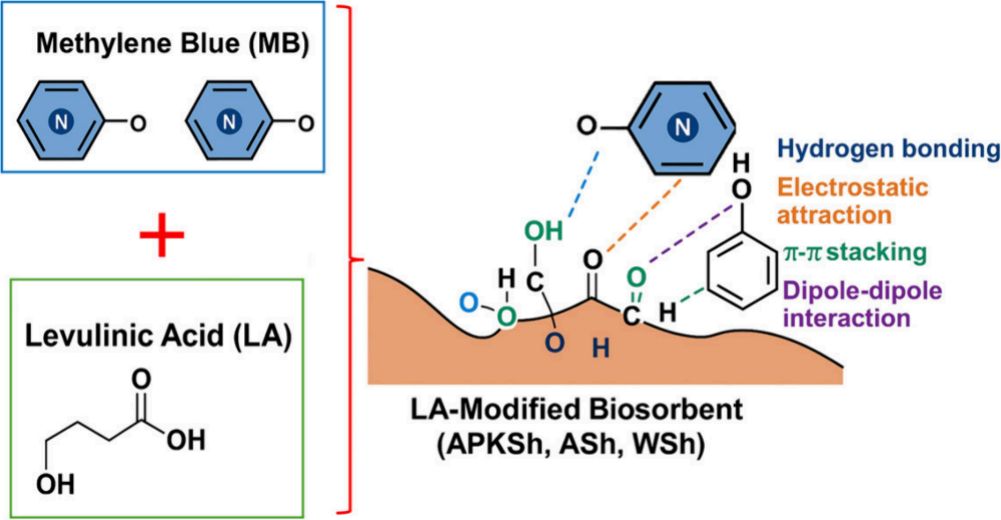
Proposed adsorption mechanism of MB on LA-modified bioadsorbents.

### Evaluation of Machine Learning Results

This study employed machine learning techniques to model and interpret the dye removal process from wastewater. Natural fruit seed wastes, including apricot, almond, and walnut shells, were ground into powder form and chemically modified with LA to develop environmentally friendly adsorbent materials. The modified adsorbents were then used to remove methylene blue (MB) dye.

The *q*_*e*_ (mg/g) and removal percentage (%) were analyzed under various parameters, including pH levels (3–10), adsorbent doses (0.4–6 g/L), initial dye concentrations (10–500 mg/L), and temperatures (25–55 °C). The data set consisted of 105 samples, each corresponding to almond, walnut, or apricot species. It included attributes like pH, adsorbent dose, dye concentration, temperature, time, and species type. The One-Hot Encoding method incorporated the species variable into the data set, and all attributes were normalized to the [0–1] range using the Min-Max normalization method.

The distributional characteristics of the entry features were analyzed both statistically and visually. The Kolmogorov–Smirnov test was applied to assess the normality of the data. It was observed that the features did not conform to a normal distribution. Furthermore, the histogram plots presented in Figure 7 demonstrate that the majority of variables manifest skewed. The presence of skewness, concentration of values in narrow ranges, and the existence of outliers, as evidenced by the histograms, provide sufficient justification for the utilization of nonparametric methodologies. Accordingly, Spearman’s rank correlation coefficient was utilized to evaluate the relevance of features.

**Figure 7:**
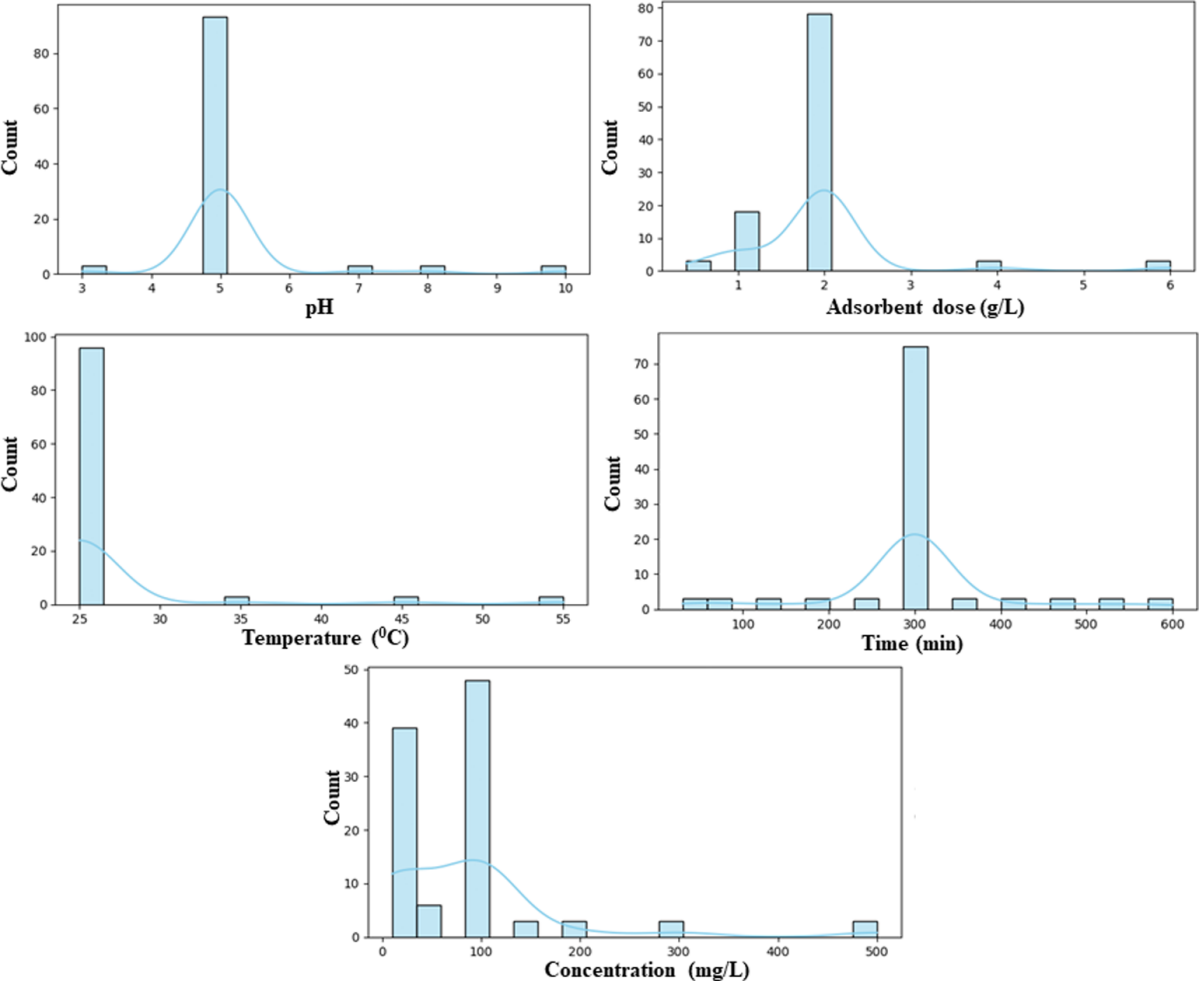
Histogram plots of features.

In this study, Spearman’s correlation analysis was employed to assess the significance of the features due to the non-normal distribution of the data set. The importance of the attributes was ranked based on their correlation with the target variables, and these rankings were corroborated by findings in the existing literature, which similarly highlight the relevance of such attributes in dye removal processes. Specifically, previous studies have consistently emphasized the critical roles of factors such as pH, adsorbent dose, and dye concentration in the adsorption process. The obtained rankings align well with prior research in the field of dye adsorption and contribute to the interpretability of the model outputs. This approach is particularly valuable for ensuring a balanced trade-off between model performance and interpretability, especially when working with limited or small-scale data sets.

Prominent machine learning algorithms, such as GB, XGB, MLP, and RF, were applied to regression estimation of adsorption capacity and removal efficiency. These algorithms were evaluated through a 5-fold cross-validation (5-fold CV) process. The highest prediction performance was achieved using the GB model, with R^2^ values of 0.8858 for removal efficiency and 0.9532 for adsorption capacity. These results demonstrate a high level of prediction accuracy, thereby confirming the effectiveness of ML-based approaches in this field. ML models are distinguished not only by their predictive accuracy but also by their ability to analyze multidimensional relationships among complex process parameters.

Recently, there has been a significant increase in the number of adsorption studies based on artificial intelligence and machine learning in the literature.^32–39^ In our study, we estimated two target variables, namely removal and adsorption capacity, using a data set consisting of three different types and machine learning methods. For our contribution to the literature, the different types were converted into separate feature columns using the one-hot encoding method during the data preprocessing stage, and the data were normalized. Additionally, correlation-based feature importance ranking was performed. Comprehensive analyses were performed using multiple machine learning models that are widely used in the literature and demonstrate high performance. Although the removal and adsorption capacity values in the data set used in the study were within a wide range, the R^2^ scores obtained demonstrate that our models successfully predicted these targets. These findings highlight the potential of machine learning to systematically and efficiently evaluate complex interactions that are often difficult to reveal through conventional experimental approaches, and to support the development of data-driven decision-making systems in chemical and environmental engineering by providing scientifically sound and practically applicable outputs for the design of sustainable wastewater treatment technologies.

This study is limited to a specific dye–biosorbent system, and the generalizability of the proposed models to other dyes or biosorbents has not been evaluated. Since adsorption behavior can vary with different chemical structures and surface properties, future work should focus on expanding the data set to include a wider range of dye types and biosorbent materials. This would allow for broader model applicability and robustness. Additionally, methods such as transfer learning or domain adaptation could be explored to enhance model generalization across different adsorption systems

## Data Availability

The data set used in this study is available via Zenodo at 10.5281/zenodo.15655679 under a Creative Commons license. The data includes experimental values of maximum adsorption capacity (*q*_*e*_) and removal percentage (%) for MB dye removal using levulinic acid-modified natural adsorbents. The data set used in this study was constructed based on the data acquisition process described by Kocaman^10^ and was reused with appropriate citation.
